# Understanding barriers to and facilitators of clinician-patient conversations about brain health and cognitive concerns in primary care: a systematic review and practical considerations for the clinician

**DOI:** 10.1186/s12875-023-02185-4

**Published:** 2023-11-06

**Authors:** Soo Borson, Gary W. Small, Quentin O’Brien, Andrea Morrello, Malaz Boustani

**Affiliations:** 1 Department of Family Medicine, Keck School of Medicine, University of Southern California, 31 E. MacArthur Crescent B414, Santa Ana, Los Angeles, CA USA; 2 Department of Psychiatry and Behavioral Sciences, University of Washington School of Medicine, Seattle, WA USA; 3 Department of Psychiatry and Behavioral Health, Hackensack Meridian School of Medicine, Hackensack, NJ USA; 4 Scientific and Medical Services, Health & Wellness Partners, LLC, Upper Saddle River, NJ USA; 5 The School of Medicine and Health Sciences, George Washington University, Washington, DC USA; 6 Division of General Internal Medicine and Geriatrics, Indiana University School of Medicine, Indianapolis, IN USA

**Keywords:** Brain health, Cognitive decline, Primary care, Dementia

## Abstract

**Background:**

Primary care clinicians (PCCs) are typically the first practitioners to detect cognitive impairment in their patients, including those with Alzheimer’s disease or related dementias (ADRD). However, conversations around cognitive changes can be challenging for patients, family members, and clinicians to initiate, with all groups reporting barriers to open dialogue. With the expanding array of evidence-based interventions for ADRD, from multidomain care management to novel biotherapeutics for early-stage AD, incorporating conversations about brain health into routine healthcare should become a standard of care. We conducted a systematic review to identify barriers to and facilitators of brain health conversations in primary care settings.

**Methods:**

We systematically searched PubMed, Scopus, Web of Science, and the Cochrane Library for qualitative or quantitative studies conducted in the US between January 2000 and October 2022 that evaluated perceptions of cognition and provider-patient brain health conversations prior to formal screening for, or diagnosis of, mild cognitive impairment or ADRD. We assessed the quality of the included studies using the Mixed Methods Appraisal Tool.

**Results:**

In total, 5547 unique abstracts were screened and 22 articles describing 19 studies were included. The studies explored perceptions of cognition among laypersons or clinicians, or provider-patient interactions in the context of a patient’s cognitive concerns. We identified 4 main themes: (1) PCCs are hesitant to discuss brain health and cognitive concerns; (2) patients are hesitant to raise cognitive concerns; (3) evidence to guide clinicians in developing treatment plans that address cognitive decline is often poorly communicated; and (4) social and cultural context influence perceptions of brain health and cognition, and therefore affect clinical engagement.

**Conclusions:**

Early conversations about brain health between PCCs and their patients are rare, and effective tools, processes, and strategies are needed to make these vital conversations routine.

**Supplementary Information:**

The online version contains supplementary material available at 10.1186/s12875-023-02185-4.

## Background

Current evidence suggests that up to 40% of Alzheimer’s disease and related dementias (ADRD) may be partly attributable to modifiable risk factors, among them hypertension, physical inactivity, hearing loss, excessive alcohol consumption, smoking, and social isolation [1, 2], and new research continues to identify others. Primary prevention efforts, such as lifestyle changes (especially if adopted as a lifespan strategy), early intervention for clinically treatable risk factors, and amelioration of social determinants of poor cognitive health could help mitigate the societal burden associated with ADRD [1–3]. For example, the Finnish Geriatric Intervention Study to Prevent Cognitive Impairment and Disability (FINGER) trial demonstrated that a multidomain lifestyle intervention could provide cognitive benefits to older people at risk for ADRD [4–6], and studies of this and similar interventions are underway in several countries.

Because of the population-wide impacts of ADRD, the US National Plan to Address Alzheimer’s Disease now includes a public health initiative [7]. The Building Our Largest Dementia (BOLD) Infrastructure for Alzheimer’s Act of 2018 authorized the Centers for Disease Control and Prevention (CDC) to establish 3 centers of excellence for ADRD prevention, early detection, and caregiving, and to energize over 40 state and local public health and other entities to develop and implement new local initiatives [8]. However, strategies for implementing and measuring the impact of such efforts in clinical practice remain ill defined, especially with respect to primary care.

Approximately 4 of 5 primary care clinicians (PCCs) consider themselves on the frontlines of brain health [9]. In the US, PCCs are usually the first point of contact for patients worried about memory loss [10] and are typically the first to detect and evaluate patients experiencing mild cognitive impairment (MCI) or ADRD [11]. As such, PCCs are uniquely positioned to initiate early conversations about brain health—even before patients have symptoms of or are diagnosed with MCI or dementia—and they are the only medical discipline capable of improving population health [12]. PCCs already play a key role in preventing several chronic diseases by offering interventions that modify risk factors [13–15], and older adults are more likely to improve their diet and physical activity when encouraged to do so by their PCC [13, 16–24].

Thus, PCCs can play a vital role in making early conversations around brain health and cognitive concerns part of routine healthcare, long before symptoms appear. Some resources offer guidance regarding interventions for brain health [25], but understanding of barriers to early conversations about brain health in the primary care setting remains limited. This systematic review summarizes the existing literature and aims to inform the development of tools, processes, and strategies that could facilitate early brain health conversations between PCCs and their patients.

## Methods

This review was conducted in accordance with the Preferred Reporting Items for Systematic reviews and Meta-Analyses (PRISMA) 2020 statement (Appendix A) [26].

All authors collaboratively identified key terms to be included in the search algorithms, and initial search strings were developed for each database using all key terms. On October 17, 2022, we searched PubMed, Scopus, Web of Science, and the Cochrane Library with predetermined algorithms using terms related to “brain health,” “cognitive dysfunction,” “cognitive impairment,” “cognitive decline,” and “dementia,” along with terms related to health knowledge, attitudes, behaviors, and communication (Appendix B). Two reviewers (QOB and AM) conducted test searches using the initial algorithms, which were then refined for each specific database to ensure a sufficient and feasible number of returned records. The first 30 titles returned by the test searches were reviewed to assess the overall relevance of the records returned by each algorithm. The initial search string appeared to be too restrictive for Scopus, Web of Science, and Cochrane Library databases; therefore, some groupings of search terms were removed from the algorithms to ensure a sufficient number of records to review.

We included studies that assessed perceptions of cognition or that evaluated PCC-patient conversations about brain health occurring before formal assessment or diagnosis of MCI/ADRD. Studies focused on evaluation, diagnosis, or treatment of MCI or ADRD were excluded. Notably, the term “Alzheimer’s” was omitted from the algorithms because our review specifically excluded studies that focused on individuals already diagnosed with ADRD. Furthermore, inclusion of “Alzheimer’s” as a term in early test searches led to an infeasible number of records to review. Other eligibility criteria included articles published in English on or after January 1, 2000. As global health systems vary widely, we limited our review to studies conducted in the US.

All retrieved references were imported into Covidence (Veritas Health Innovation, www.covidence.org). Two reviewers (QOB and AM) independently screened all titles and abstracts for relevance, and disagreements were resolved by discussion between these 2 reviewers. The same 2 reviewers independently screened all full-text articles. Additional articles were identified for inclusion through searches of the reference lists of included articles. Data were manually extracted and organized into a data charting form developed by all authors. Results were synthesized into a table summarizing key findings from each study. After the data extraction process was complete, all authors reviewed the included articles, participated in finalization of the summary table, and collaboratively identified the themes that emerged from the included articles through iterative discussion of the key findings of each paper. These discussions continued until all authors agreed on the final set of themes, at which point each article was mapped to the theme or themes that represented its key findings.

The quality of each included study was independently evaluated by 2 reviewers (QOB and AM) using the mixed methods appraisal tool (MMAT) [27]. Because we expected to include studies with a wide variety of designs in our review, we selected the MMAT as it was designed to assess the methodological quality of qualitative, quantitative, and mixed methods studies (Appendix C).

## Results

**Fig. 1:**
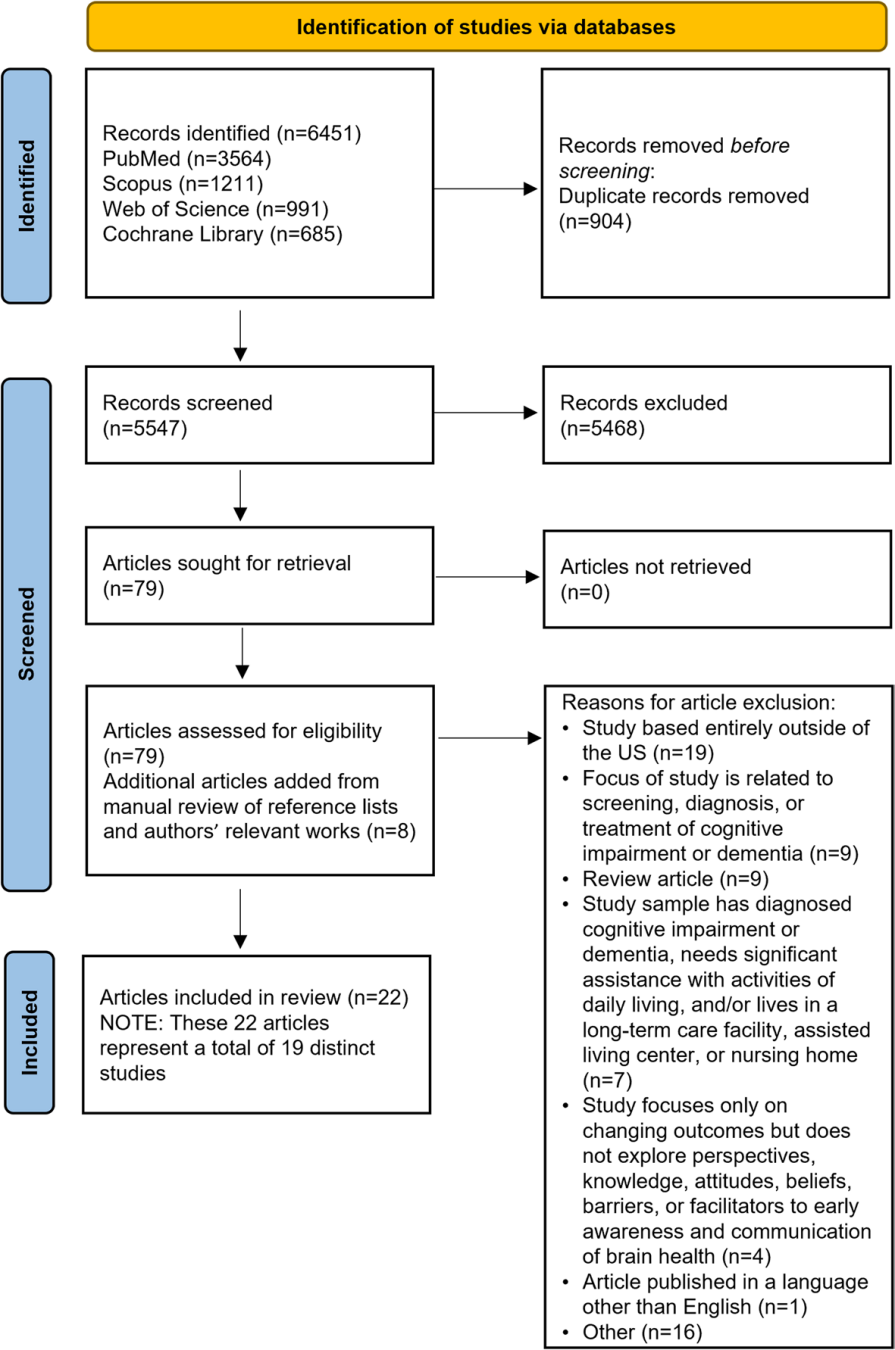
PRISMA flow diagram. PRISMA, Preferred Reporting Items for Systematic Reviews and Meta-Analyses

In total, 5547 unique records were identified. Of these, 5468 were excluded after title and abstract screening. Articles were excluded if they were published before the year 2000 or if they were published in a language other than English. Studies conducted outside the US were also excluded, as were those that focused on patients already diagnosed with Alzheimer’s disease or mild cognitive impairment, or that described specialist clinicians practicing outside of primary care settings. Additionally, review articles and studies that did not explore perspectives, knowledge, attitudes, beliefs, barriers, or facilitators to communication related to brain health or cognitive concerns were also excluded. In total, 79 full-text articles were assessed for eligibility. A manual review of reference lists and key authors’ works led to the inclusion of another 8 articles. After screening, 22 articles representing 19 unique studies were identified for inclusion (Fig. 1).

Characteristics of included studies are detailed in Table 1. Studies explored either perceptions of cognition or provider-patient interactions in the context of a patient’s cognitive complaints. We found no articles that specifically explored preventive brain health conversations. Most were descriptive in nature, using qualitative, quantitative, or mixed-methods approaches. The most common methods of data collection included focus groups, semi-structured individual interviews, surveys, or a combination. Notably, most articles (*n* = 15) were published more than 10 years ago.

**Table 1:** Characteristics of included studies

Study, Year	Method/Design	Sample	Key Findings	Themes^a^
Abdelrahman et al., 2020	Qualitative (focus groups)	Cognitively healthy adults age ≥ 55 years	• Participants voiced concerns about deteriorating cognitive health with age, including confusion around normal aging vs. cognitive decline	2, 3
Adelman et al., 2004	Mixed methods (quantitative survey data; qualitative semi-structured interviews)	Patients and caregivers age ≥ 65 years attending a first visit at a geriatric medicine outpatient clinic (*N* = 97)	• Some patients stated that they felt it is the physicians’ responsibility to raise this topic • Patients might not be aware of a cognitive problem • Physicians are more likely to raise the issue when only discussing with patient rather than patient and caregiver • Educational programs need to target those individuals who are concerned about a possible cognitive problem and are reluctant or embarrassed to discuss this issue with their physicians • Physicians may be uncomfortable raising this topic	1, 2
Corwin et al., 2009	Qualitative (focus groups)	Black/African American (*N* = 42) and White (*N* = 41) community-dwelling adults age ≥ 50 years	• Concept of aging is multidimensional • Differences in perceptions by race: Black/African Americans believe aging well means being cognitively intact, physically mobile, independent, and free from health problems; White adults described aging well in terms of living a long time, staying physically active, maintaining a positive outlook, and having good genes • The connection between physical activity and remaining independent may be a particularly motivating message for older Black/African American adults, who valued independence • Health communications focused on social involvement, and spirituality may be meaningful to all older adults	3, 4
Day et al., 2012	Cross-sectional survey	PCCs (*N* = 972)	• 40% of respondents indicated that they discuss reducing cognitive impairment often or very often in the past 6 months; 38.7% sometimes and 20% rarely • Most common advice given to maintain cognitive health: Physical activity, intellectual stimulation, healthy diet, and social activity (all > 80%) • 40% believe strength of evidence to reduce cognitive impairment is weak or very weak, 50% believe it is moderate, strong, or very strong • The most frequently reported barriers to addressing reducing cognitive impairment risk were lack of reimbursement and time (31.9%), limited scientific evidence or proven treatments (26.3%), and patients’ more immediate health issues (24.6%)	1
Friedman et al., 2009	Qualitative (focus groups)	Racially diverse group of community-dwelling adults age ≥ 50 years (*N* = 177)	• Participants indicated that they perceive a lack of information about brain health in the media • When the media does promote brain health, the focus is on diet, physical activity, brain exercises, medication, and supplements • Participants indicated opportunities for brain health education at church, field trips/outings, organized events, and existing social clubs • Participants viewed other seniors, social networks, physicians, friends/family, and TV programs as messengers for brain health education • Individual barriers to discussing cognition included participants’ other health conditions and negative attitudes toward cognitive decline • Structural barriers to discussing cognition included confusing media messages, limited information for laypersons, and perceived cost of cognitive interventions	3
Friedman et al., 2011^b^	Qualitative (focus groups)	Racially diverse group of community-dwelling adults age ≥ 50 years (*N* = 396)	• Participants identified several strategies for keeping the brain healthy and maintaining memory, including mental exercises, having a positive attitude, social interaction, physical activity, and healthy diet • Nuances in responses occurred between racial/ethnic groups due to differences in cultural beliefs • One facilitator of discussions around cognition is the consideration of social and cultural factors • Participants indicated that they perceive research around the impact of health behaviors on cognitive health to be uncertain, which presented a barrier to discussing cognition	3, 4
Friedman et al., 2013	Cross-sectional survey	Consumers (*N* = 4,728) and PCCs (*N* = 1,250)	• PCCs indicated that they advise patients to exercise, obtain intellectual stimulation, be socially active, eat a healthy diet, limit alcohol consumption, and maintain a healthy weight to prevent or delay cognitive impairment • Consumers believed that staying mentally stimulated, exercising, eating a healthy diet, maintaining a healthy weight, being socially involved, taking vitamins or dietary supplements, avoiding smoking, and taking prescribed medication could prevent or delay cognitive impairment • The majority of consumers reported that a PCC had not spoken to them about ways to stay mentally sharp in the past 12 months	3
Hochhalter et al., 2012	Qualitative (focus groups and individual interviews)	Primary care physicians (*N* = 28) and advanced practice providers (*N* = 21)	• Few providers talked about risk of cognitive impairment • When they discussed cognitive impairment, it was in the context of the benefits of physical activity on vascular risk, cancer prevention strategies, or screening for risky behaviors • Advice given to patients to maintain cognitive health included staying busy, volunteering, being socially engaged, being physically active, eating healthfully, doing puzzles or games, reading, learning new things or trying new activities, and disease management of other medical conditions • Barriers to discussing cognitive health included both system-level issues (e.g., lack of time, lack of a relationship with a patient) and patient-level issues (e.g., nonadherence to PCC recommendations for behavior change)	1, 2
Laditka et al., 2009^b^	Qualitative (focus groups)	Racially diverse group of community-dwelling adults age ≥ 50 years (*N* = 396)	• Participants described their views on aging well as they relate to cognitive health • Views on aging well differed by race and ethnicity • Discussions of cognition should include both culturally sensitive messages and broad messages that apply to many racial and ethnic groups to promote cognitive health	3, 4
Laditka et al., 2011^b^	Qualitative (focus groups)	Racially diverse group of community-dwelling adults age ≥ 50 years (*N* = 396)	• Participants discussed their concerns about the ability to keep their memory or ability to think as they age; concerns included memory loss, forgetfulness, becoming a “burden,” stigma, and behavioral changes associated with cognitive decline • Concerns differed by race and ethnicity and geographic location • It may be difficult to convince those to participate in health behaviors that reduce their risk of cognitive decline if the evidence supporting these health behaviors is perceived as uncertain	2, 3
Laditka et al., 2012	Qualitative (focus groups)	Filipino Americans who provide care to individuals with dementia (*N* = 25)	• Participants indicated that they saw social engagement, leisure, healthy diets, and avoiding smoking, alcohol, and drugs as beneficial for cognitive health • Authors highlighted that Filipino Americans are more likely to work in health professions and as paid caregivers, which may have an influence on beliefs and attitudes related to cognitive health • Facilitators of conversations around cognition include culturally relevant messaging and educational materials provided in native languages • Media sources and community centers (e.g., churches) can be used to disseminate messaging	3, 4
Light et al., 2022	Qualitative (individual interviews)	Spanish-speaking immigrants age ≥ 60 years (*N* = 30)	• Participants perceived healthy aging as including maintaining independence, memory, emotions, and orientation • Participants indicated that physical, social, and cognitive engagement is important to care for the brain • Participants’ knowledge about cognition and brain health came from communities, healthcare settings, and the media • Clear and concise messaging is a facilitator of conversations about cognition • Collaborations between healthcare providers, community centers, community classes, churches or other religious centers, and media outlets can help disseminate brain health information	3, 4
Mace et al., 2022	Qualitative (focus groups) with a virtual open pilot study	Adults age ≥ 60 years with subjective cognitive decline who were interested in changing ≥ 1 modifiable risk factor (*N* = 11)	• Participants understood aging and biomedical risk factors for dementia • Participants acknowledged the importance of exercise, mental stimulation, and nutrition for promoting brain health • Participants did not acknowledge the importance of sleep, socializing, and moderation of alcohol or substance use for maintaining brain health • Participants were interested in mindfulness-based lifestyle interventions	3
Olscamp et al., 2019	Qualitative (focus groups)	Informal caregivers of people with AD (*N* = 10)	• Participants reported that they had limited exposure to information about physical activity and brain health (i.e., not hearing about the connection between physical activity and brain health in the media) • Participants were concerned with the consistency and reliability of the evidence to support physical activity for brain health • Participants were more likely to trust information from credentialed and licensed professionals (e.g., physicians, therapists) • Facilitators of discussing cognition included using evidence-based information, fostering trust in licensed providers, and delivering consistent messages	3
Onafraychuk et al., 2021	Cross-sectional survey	Adults age ≥ 18 years (*N* = 169)	• Participants indicated that they would be willing to invest time in activities to maintain brain health and cognition if they saw those activities as being efficacious • Brain training and aerobic exercise were seen as efficacious, while meditation was seen as less efficacious • Participants’ anticipated enjoyment of an activity was a predictor of their willingness to engage in the activity • Individual limitations (e.g., mobility limitations, physical ailments, etc.) may hinder an individual from discussing cognition or taking steps to maintain brain health	3
Price et al., 2011	Qualitative (focus groups) and a cross-sectional survey	Black and White adults age 65–74 years (*N* = 55)	• In general, participants understood the connection between physical activity and cognitive health, but they felt that evidence supporting the connection was lacking • White men expressed less concern about cognitive decline compared to other participants • Black women expressed spiritual undertones in focus groups, highlighting potential benefits of faith-based messages	3, 4
Sharkey et al., 2009	Qualitative (focus groups) and a cross-sectional survey	Spanish-speaking Mexican American adults age ≥ 55 years (*N* = 33)	• Participants described aging well as “staying right in the mind” • Not aging well was seen as being lost or “closed off” from other people; there was a possible supernatural malicious element to not aging well related to views on spirituality • Participants’ concerns about aging and memory included being alone in the world and worries about becoming a burden to others • Participants indicated that mental and physical activities contribute to aging well by distracting from “bad thoughts” • Authors highlighted that the results from this study contrasted with a similar study that included English-speaking Hispanic focus groups on the same topic, attributing these differences to language as well as household income, education, neighborhood deprivation, and degree of assimilation	3, 4
Warren-Findlow et al., 2010	Qualitative (focus groups and individual interviews) and a cross-sectional survey	PCCs, including physicians (*N* = 28) and advanced practice providers (*N* = 21)	• Participants indicated that online sources, popular media, and continuing medical education were their most common sources of information about cognitive health • Participants were concerned about inconclusive research on cognitive health (i.e., “The brain is still pretty much a black box”) • Popular media can be both a barrier to and facilitator of discussions of brain health between providers and patients • Having a network of specialists connected to PCCs can facilitate knowledge transfer and improve brain health understanding among providers • Inconclusive brain health research leaves PCCs ill-equipped for these conversations	1, 2, 3
Weiner-Light et al., 2021	Qualitative (individual interviews	Spanish-speaking Latin American immigrants age ≥ 60 years (*N* = 30)	• Participants indicated that spirituality and their relationship with God forms the basis of healthy aging and maintaining health in older age • Authors highlighted the potential utility of customized spiritual interventions to increase the effectiveness of brain health promotion efforts among Latin American immigrants	4
Wilcox et al., 2009^b^	Qualitative (focus groups)	Racially diverse group of community-dwelling adults age ≥ 50 years (*N* = 396)	• Participants indicated that there is a positive link between physical activity and dietary practices and brain health • Different ethnic groups expressed desire to do different types of physical activity • Different ethnic groups emphasize the importance of different characteristics for a healthy diet • Differences among groups may be attributable to differences in acculturation	3
Wu et al., 2009	Qualitative (focus groups)	Community-dwelling adults age ≥ 55 years (*N* = 67)	• Participants expressed some confusion regarding AD vs. normal aging, and they identified underlying causes of dementia as well as preventive strategies for avoiding dementia in later life (e.g., social engagement, physical activity, etc.) • Participants expressed reluctance to address brain health because people who use preventive strategies can still develop AD anyway • Women were more likely than men to take the lead in providing healthcare information for their families • Women were more concerned with difficulty of preparing healthy food while men cited taste preferences, fast-food convenience, and lack of self-control • Women endorsed social/physical activities like group exercise classes while men discussed manual labor and employment in relation to physical exercise • Gender differences may be due to cultural traditional gender roles seen in this age cohort	3, 4
Zhai et al., 2022	Qualitative (focus groups)	Korean, Samoan, Cambodian, and Chinese Americans age ≥ 50 years (*N* = 62)	• The majority of participants assumed that memory loss was part of normal aging • Participants were eager to learn the causes of memory loss and dementia and evidence-based practices to delay memory decline • Not all Asian Americans and Pacific Islander individuals have the same understanding of memory loss; differences exist across and within cultures • Different views from each ethnic group might be formed by both cultural beliefs and by social and structural factors that each ethnic group uniquely faces during immigration	2, 3, 4

*Abbreviations*: *AD *Alzheimer’s disease, *PCC *Primary care clinician

^a^These four themes are: (1) PCCs are hesitant to discuss brain health and cognitive concerns (2). Patients are hesitant to raise cognitive concerns (3). Evidence to guide clinicians in developing treatment plans that address cognitive decline is often poorly communicated (4). Social and cultural context influence perceptions of brain health and cognition, and therefore affect clinical engagement

^b^These papers represent separate analyses from the same study

The majority of articles (*n* = 20) included lay participants (e.g., patients, caregivers, and community members); 4 included PCCs (e.g., physicians, nurse practitioners, and physician assistants). One study included both laypersons and PCCs [28]. Several studies specifically explored the beliefs, attitudes, and knowledge of US racial and ethnic minority groups: 2 each were conducted with Black/African American [29, 30] or Asian American participants [31, 32], 3 with Latino participants [33–35], and 2 with racially and ethnically diverse participants [36–40].

Overall, the quality of the studies was high. Using the MMAT to appraise the quality of the studies, we found that 15 of the 16 qualitative studies adequately met all criteria for methodological quality. [29–33, 35–44] Quality was more varied among quantitative descriptive studies [28, 45, 46], and the lowest quality studies included in this review used mixed methods designs [34, 47, 48]. Detailed quality assessment results can be found in Appendix C.

Our review uncovered 4 main themes that provide insight into barriers to and facilitators of implementing early conversations about brain health between PCCs and their patients.

### Theme 1: PCCs are hesitant to discuss brain health and cognitive concerns

Studies addressed discussions of cognitive concerns or impairment, rather than brain health as a general category of health or wellness. Many PCCs are uncomfortable discussing cognitive concerns with their patients, or lack resources and support for these conversations [47]. In a survey of 972 physicians, approximately half of whom were family or general practitioners, 31.9% of respondents reported that lack of reimbursement was the most frequent barrier to discussing cognitive impairment with patients [45]. Other barriers commonly reported by respondents included a lack of proven treatments and limited scientific evidence for prevention of MCI or ADRD (26.3%) and the need to address more pressing medical issues (24.6%) [45]. A qualitative study of 49 PCCs reported additional barriers to talking about cognition, such as a lack of time during appointments, therapeutic nihilism, insufficient evidence regarding prevention and treatment of cognitive impairment, and negative patient attitudes about cognition education [42]. Although these studies were conducted before the 2011 introduction of the Annual Wellness Visit, a preventive Medicare benefit that requires identification of any existing cognitive impairment [49], there is little evidence to suggest that PCCs’ attitudes and practices have dramatically changed.

According to the articles included in the present review, addressing these barriers would require resolving constraints related to time, clinic resources, and reimbursement; tailoring education to improve PCCs’ comfort and cultural awareness around discussing cognitive difficulties and interventions with their patients; and increasing their confidence in the evidence supporting the value of interventions. In a study of PCCs across 3 states, practitioners reported using a variety of sources, such as continuing medical education, popular media, and online resources to educate themselves on brain health [48], a finding corroborated by another study [45]. Effective dissemination of educational material related to brain health, cognition, and cognitive disorder care can and should occur through multiple approaches (e.g., online sources, continuing medical education, and professional journals). Strategies for establishing effective collaboration between PCCs and cognitive disorder specialists—who are scarce in many regions—have not yet been described in the literature. Productive areas for exploration include how best to facilitate knowledge transfer, how to provide PCCs with up-to-date understanding of interventions usable in primary care settings [48], and how best to define the clinical contributions of generalists and specialists in the evaluation and care of people with cognitive impairments.

### Theme 2: patients are hesitant to raise cognitive concerns

In a study of older, primarily White patients attending a first visit to an outpatient geriatric practice, patients identified several barriers to discussing cognitive concerns with their physician [47]. Most often, patients felt it was the physician’s responsibility to initiate the discussion. Others intended to initiate the conversation during the appointment but forgot. In this same study, researchers noted that some patients may hesitate to raise cognitive concerns due to feelings of embarrassment or shame, resulting from stigma surrounding MCI and ADRD [47]. The role of stigma was also reported in a study of ethnically diverse older adults [38]. In that study, 42 focus groups were conducted with older adults representing 6 racial and ethnic groups (i.e., African American, Native American, Chinese American, Latino, White, and Vietnamese American). Researchers found that Native American, Chinese American, and Vietnamese American respondents were specifically concerned about stigma associated with MCI or ADRD and how stigma may impact their family relationships [38]. Two other studies suggested that individuals may not distinguish normal aging from cognitive decline [32, 41]. For example, in a study of 62 older Asian American participants, 95% of participants assumed that memory loss was part of normal aging [32].

The studies included in the present review also highlighted the need for solutions that address patients’ hesitance to initiate important brain health discussions and provided examples of such solutions. For instance, if patients believe it is the responsibility of their PCC to start discussions regarding cognition and if patients are likely to forget to raise cognitive concerns during appointments, then PCCs can remove this barrier by taking the lead [47]. However, as mentioned previously, PCCs are also often hesitant to start these conversations [42, 45, 47, 48]. One approach to successful, practical implementation is to treat discussions of cognitive concerns as routine by embedding questions around cognition within a medical review of systems [47]; however, additional resources are clearly needed to aid clinicians in leading these conversations.

### Theme 3: evidence to guide clinicians in developing treatment plans that address cognitive decline is often poorly communicated

Both lay media and scientific discussions of brain health are often confusing, contradictory, or limited [30, 36–38, 48]. Thus, although most people recognize the importance of aging well and maintaining brain health [28, 30–34, 36, 37, 39–41, 48, 50], many remain skeptical of brain health research and unsure about which cognitive interventions are worthwhile, leading to therapeutic nihilism [43, 44, 46]. This theme was common across multiple studies reviewed. To date, no study has addressed whether the perception of ADRD as a disease that affects only elderly individuals may have promoted catastrophic thinking and reinforced avoidance on the part of both clinicians and their patients. In addition, many publications have described ADRD as a “terminal illness” rather than a manageable chronic condition [51–53]. Although this characterization may have been intended to elevate the importance of ADRD in public and medical discourse, this portrayal may have reinforced fear and avoidance.

To facilitate action around brain health, the studies included in the present review suggest that both laypersons and PCCs should be educated on the existing evidence that supports a range of interventions for brain health and cognitive disorders [29, 50]. This education should be communicated clearly, concisely, and consistently [30, 33, 43].

### Theme 4: social and cultural context influence perceptions of brain health and cognition, and therefore affect clinical engagement

Several studies found that differences in beliefs about brain health and cognitive decline among racial and ethnic minority groups could be attributed to cultural differences. For example, in one study, Black people—and particularly Black women—were more likely to use language expressing spiritual elements when discussing brain health and concerns about cognitive decline; this fact highlights the importance of recognizing differences in the way individuals frame their concerns; one study suggests that Black women may respond more positively to messages that incorporate spiritual ideas [30]. Similarly, in 2 other studies, Latin American participants expressed spiritual and supernatural ideas related to brain health and cognitive decline [34, 35]. Both studies highlighted the value of collaborating with faith-based organizations to more effectively tailor messages for Latin American populations, and emphasized references to prayer, church-going, gratitude to a higher power, and a connection with God [35]. One study found beliefs and knowledge about memory loss differed across and within Asian American and Pacific Islander (AAPI) participants, and these beliefs were informed by the unique social and structural factors of each AAPI ethnic group [32]. Immigration status and language barriers among various AAPI groups were also found to limit opportunities to engage in social and economic activities in the US, leading to a need for messaging that is not only culturally sensitive but that can also help surmount barriers to accessing appropriate health care [32]. On the other hand, another study found that Filipino American individuals often have a strong biomedical understanding of brain health through workplace exposure to individuals with cognitive impairment; many are employed in healthcare professions, including in long-term care [31].

Among laypersons, sex and gender may also play a role in conceptions of brain health and behaviors to maintain or improve brain health. Women often take the lead in providing healthcare information for their families, highlighting the need to specifically engage this population in early conversations on brain health [44]. Societally influenced traditional gender roles also appeared to affect specific beliefs and behaviors related to cognition. For example, while both women and men understood the importance of physical exercise and social engagement for maintaining cognitive functioning, women endorsed social and physical activities like group exercise classes, whereas men indicated that manual labor and formal employment could fulfill physical exercise and social needs [44].

The studies included in the present review also highlight that conversations around cognition should be tailored to specific patients and audiences, include culturally relevant information, and consider both the social and cultural contexts in which patients live [29–31, 37, 39]. The literature also reveals that primary care is just one setting in which to circulate this information; partnering with local communities’ trusted media sources and institutions is important for disseminating brain health messages [31, 33].

## Discussion

Our systematic review revealed 4 themes describing barriers to and facilitators of conversations around brain health in primary care: (1) PCCs are hesitant to discuss brain health and cognitive concerns; (2) patients are hesitant to raise cognitive concerns; (3) evidence to guide clinicians in developing treatment plans that address cognitive decline is often poorly communicated; and (4) social and cultural context influence perceptions of brain health and cognition, and therefore can affect clinical engagement.

Although PCCs’ and patients’ hesitation to discuss brain health and cognition was identified in literature from over a decade ago, this hesitation still looms large in clinical practice today. A recent report by the Alzheimer’s Association found that although 75% of PCCs provide direct care for patients with MCI or ADRD, many are uncomfortable diagnosing ADRD, and the vast majority had little to no residency training in ADRD diagnosis [54]. Additionally, stigma [38] and the perception that ADRD interventions are not available or effective [43, 44, 46, 55] make these discussions challenging. These perceptions may be partially due to the need for multimodal rather than singular interventions to effectively address and potentially slow cognitive decline [56].The recent approval of AD disease-modifying therapies may lend new impetus for these conversations by decreasing therapeutic nihilism.

Important innovations in clinician and health system training are occurring now. For example, the Gerontological Society of America has developed the Kickstart, Assess, Evaluate, Refer (KAER) Toolkit for Primary Care Teams to provide practical tools, processes, and strategies for PCCs who wish to initiate conversations about brain health, detect and diagnose ADRD, and provide patients with community-based support [57]. The KAER model identifies 4 broad steps to achieve greater awareness of brain health (“Kickstart”), increase detection of cognitive impairment (“Assess”), initiate earlier diagnostic evaluation (“Evaluate”), and refer people with ADRD (“Refer”). The “Kickstart” phase includes practical recommendations for initiating conversations around brain health and cognitive concerns. These recommendations include raising the topic of brain health during initial patient visits, asking patients about their memory and cognition, and making these discussions part of routine care, among others.

The BOLD Public Health Center of Excellence on Early Detection of Dementia (EDD) has developed a basic toolkit for health systems [58]. Like the KAER model, the BOLD toolkit provides practitioners with practical guidance for having conversations with patients before performing formal dementia screening. The BOLD toolkit emphasizes ways to build trust, use positive framing to normalize conversations about cognition in health care settings, and be ready with simple statements that explain the importance of cognition in everyday functioning.

**Fig. 2:**
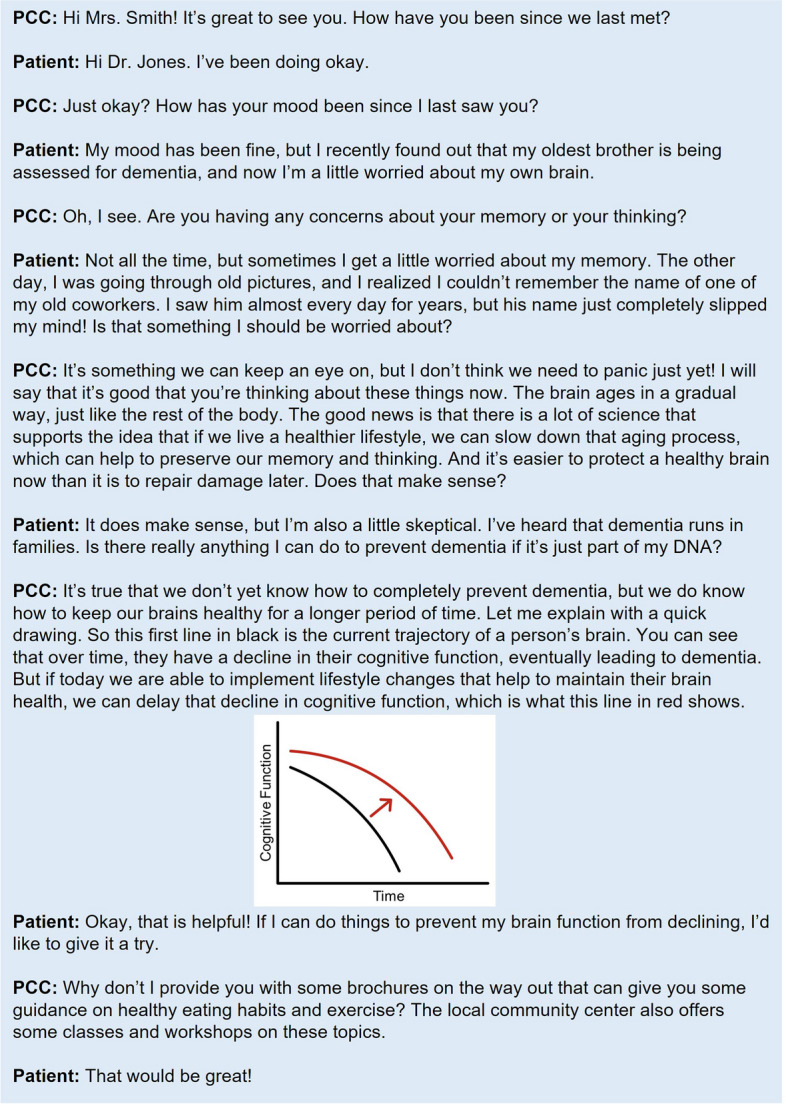
An example of initiating conversations around brain health and cognition with a patient who has cognitive concerns. *PCC, primary care clinician*

**Fig. 3:**
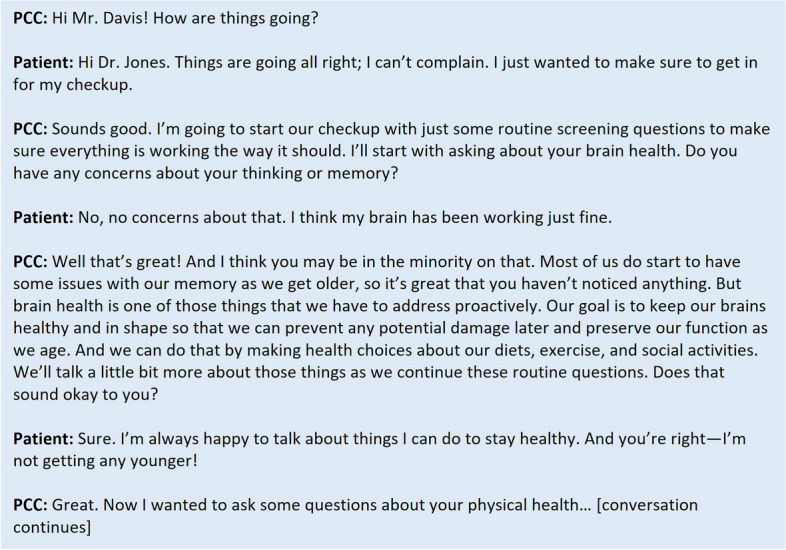
An example of initiating conversations around brain health and cognition with a patient who does not express cognitive concerns. *PCC, primary care clinician*

Although resources like the KAER model and the BOLD EDD toolkit provide practical strategies that can be adapted to meet the needs of PCCs and their patients, evidence regarding their use in clinical practice is undeveloped. To further assist PCCs, Figs. 2 and 3 provide examples of hypothetical conversations about cognition, initiated by a PCC, both with patients who do and do not express concerns about cognitive decline. Both conversations incorporate elements from the KAER and BOLD toolkits, starting with raising the topic of brain health and normalizing the conversation as part of a routine healthcare visit. After specifically asking the patient if they have any memory or cognition concerns, the PCC builds trust by listening and responding to the patient’s concerns, offering guidance that reassures the patient that the PCC is a trusted source of information on brain health. Finally, the PCC encourages the brain health conversation to continue by providing tangible resources and explaining how brain health fits into a larger picture of overall health.

These conversations are quick but informative and allow PCCs to incorporate conversations about brain health into routine visits, even when no cognitive concern exists. Building basic brain health awareness as part of primary care is an important foundation for preventive interventions, when possible, and identifying decline when it does occur. When cognitive impairment is suspected, the language and content of these conversations should be tailored to the patient’s social and cultural context. For example, nurse-led, faith-based, culturally tailored educational programs about ADRD and early detection have generally been positively received by members of the Black community [59]. Although this education is provided in a group setting instead of in an individual encounter, the culturally relevant messaging developed as part of these programs can serve as a guide for physicians. This point is particularly important given how stigma and sociocultural differences can negatively impact health-seeking behaviors related to cognitive concerns and discourage inclusion in clinical research and advances in clinical care [60–65]. Starting conversations in individuals who are at risk because of age, comorbid conditions, or known risk factors—but are still cognitively normal—may increase a patient’s trust and openness, as well as the clinician’s comfort in initiating discussion should cognitive symptoms start to appear.

In addition to the resources and strategies that can help guide provider-patient conversations about brain health, there is also a need for a system-level approach to implementation that creates demand for these provider-patient conversations. The Agile processes (i.e., Agile Innovation, Agile Implementation, and Agile Diffusion) provide a framework for facilitating the rapid uptake and diffusion of evidence-based solutions [66, 67]. The Agile framework has been used to guide the implementation and evaluation of evidence-based interventions for dementia care [68], and the Agile principles can also be applied to early conversations around brain health in the primary care setting. One concept used within the Agile processes is the “nudge,” which refers to a small change in environment that can positively influence individuals’ behaviors and choices. A simple and easily implemented strategy could be a poster on a clinic wall encouraging patients to ask questions about their cognition. Another key component of the approach is the idea of creating market demand for an evidence-based intervention prior to rollout and scale-up within an organization. In the context of discussions about brain health, healthcare policies may play a role in driving this demand. As one example, in 2011, Medicare established the Annual Wellness Visit, which requires providers to discuss cognition and cognitive concerns with their Medicare patients [49]. Although the literature has not conclusively determined the effectiveness of the Annual Wellness Visit in improving dementia diagnosis [69–71], this policy feature provides an avenue for incentivizing brain health conversations. We recommend considering Agile processes and concepts such as nudges and market demand when looking to implement early conversations about cognition at the practice or system level.

This systematic review is subject to several limitations. Only English-language studies conducted in the US were included, so some relevant literature was excluded by design during the screening process. Studies assessing conversations with patients formally screened for or diagnosed with MCI or ADRD were also excluded, though the results of these studies may also contain valuable information that can be applied earlier in a patient’s journey. However, many patients are unaware they have been diagnosed with MCI or ADRD [72, 73], which means that the studies we reviewed may have included patients who qualify for a formal diagnosis. In addition, a majority of the included articles were published more than 10 years ago, presenting another limitation and highlighting a gap in the literature. This gap presents an opportunity to conduct research on barriers and facilitators to early conversations around brain health and cognitive concerns in primary care settings. Such research should include implementation research to evaluate the real-world effectiveness of interventions that aim to mitigate barriers and optimize facilitators to these important conversations.

## Conclusions

In this systematic review we sought to identify barriers to and facilitators of early conversations around brain health and cognitive concerns between PCCs and their patients before patients receive a formal screening or diagnosis of MCI or ADRD. Findings from this review revealed that both PCCs and patients are hesitant to initiate these conversations, evidence to inform brain health interventions is often poorly communicated, and social and cultural factors impact clinical engagement between PCCs and their patients.

These themes highlight the importance of framing discussions about brain health and cognitive concerns as part of routine primary care, clearly translating knowledge about the effectiveness of brain health interventions to clinicians in order to reduce therapeutic nihilism, and partnering with communities to tailor education to patients’ social and cultural contexts. Putting these key suggestions within the context of the broader literature also underscores the importance of implementing system-level approaches to facilitate these conversations between PCCs and their patients. Future research should identify additional barriers that hinder early conversations about brain health and cognition between PCCs and their patients and evaluate the effectiveness and feasibility of both interpersonal and system-level approaches to address these barriers.

### Supplementary Information

**Additional file 1: Appendix A.** PRISMA 2020 Checklist

**Additional file 2: Appendix B.** Database Search Algorithms

**Additional file 3: Appendix C.** Mixed Methods Appraisal Tool (MMAT) Quality Assessment

## Data Availability

All data generated or analyzed during this study are included in this published article (and its supplementary information files).

## Abbreviations

AAPI: Asian American and Pacific Islander
ADRD: Alzheimer’s disease and related dementias
BOLD: Building Our Largest Dementia Infrastructure for Alzheimer’s Act
CDC: Centers for Disease Control and Prevention
EDD: Early Detection of Dementia
FINGER: Finnish Geriatric Intervention Study to Prevent Cognitive Impairment and Disability trial
KAER: Kickstart, Assess, Evaluate, Refer
MMAT: Mixed methods appraisal tool
MCI: Mild cognitive impairment
PCC: Primary care clinician
PRISMA: Preferred Reporting Items for Systematic reviews and Meta-Analyses
