# Supplementary material for: Understanding barriers to and facilitators of clinician-patient conversations about brain health and cognitive concerns in primary care: a systematic review and practical considerations for the clinician
Source: BMC Prim Care. 2023 Nov 6;24:233. doi: 10.1186/s12875-023-02185-4 (PMC10626639; doi:10.1186/s12875-023-02185-4)
Supplement: Supplementary file 2 — Additional file 2: Appendix B. Database Search Algorithms [file 12875_2023_2185_MOESM2_ESM.docx]

**Appendix B.** Database Search Algorithms

| **Database** | **Algorithm** |
| --- | --- |
| PubMed | (cognitive dysfunction / diagnosis[TIAB] OR cognitive dysfunction / epidemiology[TIAB] OR cognitive dysfunction / prevention & control[TIAB] or cognition*[TIAB] OR cognitive impairment / diagnosis[TIAB] OR cognitive impairment / epidemiology[TIAB] OR cognitive impairment / prevention & control[TIAB] OR cognitive decline / diagnosis[TIAB] OR cognitive decline / epidemiology[TIAB] OR cognitive decline / prevention & control[TIAB] OR dementia / diagnosis*[TIAB] OR dementia / epidemiology[TIAB] OR neurocognitive disorders / prevention & control[TIAB] OR brain[TIAB] OR “brain health”[TIAB]) AND (preference* OR belief* OR attitude* OR choice behavior* OR communication* OR conversation* OR counseling* OR health education* OR health knowledge, attitudes, practice* OR health promotion* OR information dissemination* OR language* OR life style* OR knowledge sharing* OR motivation* OR office visits* OR primary health care* OR risk factors* OR risk reduction behavior* OR barriers* OR facilitators*) AND (focus groups*[TIAB] OR health surveys*[TIAB] OR interviews*[TIAB] OR qualitative research*[TIAB] OR surveys*[TIAB] OR questionnaires*[TIAB]) |
| Scopus | (TITLE-ABS ( "cognitive dysfunction" ) OR TITLE-ABS ( "cognitive impairment" ) OR TITLE-ABS ( "cognitive decline" ) OR TITLE-ABS ( "brain health" ) OR TITLE-ABS ( "brain decline" ) ) AND (TITLE-ABS ( "primary care" ) OR TITLE-ABS ( "primary health care" ) ) |
| Web of Science | (TI= ("cognitive dysfunction" ) OR AB=(“cognitive dysfunction”) OR TI=("cognitive impairment") OR AB=("cognitive impairment") OR TI=("cognitive decline") OR AB=("cognitive decline") OR TI=("brain health") OR AB=("brain health") OR TI=("brain decline") OR AB=("brain decline")) AND (TI=("primary care") OR AB=("primary care") OR TI=("primary health care") OR AB=("primary health care")) |
| Cochrane Library | (cognitive):ti,ab,kw OR (cognition):ti,ab,kw OR (“brain health”):ti,ab,kw OR (“brain decline”):ti,ab,kw |
