# Supplementary material for: Understanding barriers to and facilitators of clinician-patient conversations about brain health and cognitive concerns in primary care: a systematic review and practical considerations for the clinician
Source: BMC Prim Care. 2023 Nov 6;24:233. doi: 10.1186/s12875-023-02185-4 (PMC10626639; doi:10.1186/s12875-023-02185-4)
Supplement: Supplementary file 3 — Additional file 3: Appendix C. Mixed Methods Appraisal Tool (MMAT) Quality Assessment [file 12875_2023_2185_MOESM3_ESM.docx]

**Appendix C. Mixed Methods Appraisal Tool (MMAT) Quality Assessment**

| **1st Author, Year** | **Methodological Quality Criteria** | | | | | | |
| --- | --- | --- | --- | --- | --- | --- | --- |
| **Qualitative** | | | | | | | |
|  | Are there clear research questions? | Do the collected data address the research questions? | Is the qualitative approach appropriate to answer the research question? | Are the qualitative data collection methods adequate to address the research question? | Are the findings adequately derived from the data? | Is the interpretation of results sufficiently substantiated by data? | Is there coherence between qualitative data sources, collection, analysis and interpretation? |
| Abdelrahman et al., 2020 | ✓ | ✓ | ✓ | ✓ | ✓ | ✓ | ✓ |
| Corwin et al., 2009 | ✓ | ✓ | ✓ | ✓ | ✓ | ✓ | ✓ |
| Friedman et al., 2009 | ✓ | ✓ | ✓ | ✓ | ✓ | ✓ | ✓ |
| Friedman et al., 2011^a^ | ✓ | ✓ | ✓ | ✓ | ✓ | ✓ | ✓ |
| Hochhalter et al., 2012 | ✓ | ✓ | ✓ | ✓ | ✓ | ✓ | ✓ |
| Laditka et al., 2009^a^ | ✓ | ✓ | ✓ | ✓ | ✓ | ✓ | ✓ |
| Laditka et al., 201^a^ | ✓ | ✓ | ✓ | ✓ | ✓ | ✓ | ✓ |
| Laditka et al., 2012 | ✓ | ✓ | ✓ | ✓ | ✓ | ✓ | ✓ |
| Light et al., 2022 | ✓ | ✓ | ✓ | ✓ | ✓ | ✓ | ✓ |
| Mace et al., 2022^b^ | ✓ | ✓ | ✓ | ✓ | Can’t tell | Can’t tell | Can’t tell |
| Olscamp et al., 2019^c^ | ✓ | ✓ | ✓ | ✓ | ✓ | ✓ | ✓ |
| Price et al., 2011^d^ | ✓ | ✓ | ✓ | ✓ | ✓ | ✓ | ✓ |
| Weiner-Light et al., 2021 | ✓ | ✓ | ✓ | ✓ | ✓ | ✓ | ✓ |
| Wilcox et al., 2009^a^ | ✓ | ✓ | ✓ | ✓ | ✓ | ✓ | ✓ |
| Wu et al., 2009 | ✓ | ✓ | ✓ | ✓ | ✓ | ✓ | ✓ |
| Zhai et al., 2022 | ✓ | ✓ | ✓ | ✓ | ✓ | ✓ | ✓ |
| **Quantitative randomized controlled trials** | | | | | | | |
|  | Are there clear research questions? | Do the collected data address the research questions? | Is randomization appropriately performed? | Are the groups comparable at baseline? | Are there complete outcome data? | Are outcome assessors blinded to the intervention provided? | Did the participants adhere to the assigned intervention? |
| No studies identified | N/A | N/A | N/A | N/A | N/A | N/A | N/A |
| **Quantitative non-randomized** | | | | | | | |
|  | Are there clear research questions? | Do the collected data address the research questions? | Are the participants representative of the target population? | Are measurements appropriate regarding both the outcome and intervention (or exposure)? | Are there complete outcome data? | Are the confounders accounted for in the design and analysis? | During the study period, is the intervention administered (or exposure occurred) as intended? |
| No studies identified | N/A | N/A | N/A | N/A | N/A | N/A | N/A |
| **Quantitative descriptive** | | | | | | | |
|  | Are there clear research questions? | Do the collected data address the research questions? | Is the sampling strategy relevant to address the research question? | Is the sample representative of the target population? | Are the measurements appropriate? | Is the risk of nonresponse bias low? | Is the statistical analysis appropriate to answer the research question? |
| Day et al., 2012 | ✓ | ✓ | ✓ | Can’t tell | Can’t tell | Can’t tell | ✓ |
| Friedman et al., 2013 | ✓ | ✓ | ✓ | Can’t tell | Can’t tell | Can’t tell | ✓ |
| Onafraychuk et al., 2021 | ✓ | ✓ | Can’t tell | Can’t tell | ✓ | X | ✓ |
| **Mixed methods** | | | | | | | |
|  | Are there clear research questions? | Do the collected data address the research questions? | Is there an adequate rationale for using a mixed methods design to address the research question? | Are the different components of the study effectively integrated to answer the research question? | Are the outputs of the integration of qualitative and quantitative components adequately interpreted? | Are divergences and inconsistencies between quantitative and qualitative results adequately addressed? | Do the different components of the study adhere to the quality criteria of each tradition of the methods involved? |
| Adelman et al., 2004 | ✓ | ✓ | X | X | X | ✓ | ✓ |
| Sharkey et al., 2009 | ✓ | ✓ | X | X | X | Can’t tell | Can’t tell |
| Warren-Findlow et al., 2010 | ✓ | ✓ | X | X | X | Can’t tell | ✓ |

✓ = Yes; X = No.

^a^These papers represent separate analyses from the same study.

^b^This article described the results from 2 separate studies. The first study involved qualitative focus groups that assessed participants’ knowledge of brain health and dementia prevention; this first study was included in our review. The second study was a pilot program that aimed to assess feasibility of a mindfulness program, and therefore it did not assess items that were of interest to the current systematic review. Based on the differing objectives of these 2 studies, only the first qualitative study was included in our analysis.

^c^This study was composed of 2 parts, the first being a content analysis of documents from national physical activity organizations and the second being an exploration of brain health knowledge and beliefs among informal caregivers. Given the parameters of this systematic review, only the second part of the study was included, and therefore this study was assessed as a qualitative study.

^d^While the authors of this study describe a quantitative survey component, the results of said survey are not included in the results or the interpretation of those results. Therefore, this study was assessed as a qualitative study.
